# Effects of two zinc supplementation levels and two zinc and copper sources with different solubility characteristics on the growth performance, carcass characteristics and digestibility of growing‐finishing pigs

**DOI:** 10.1111/jpn.13447

**Published:** 2020-09-23

**Authors:** Sandra Villagómez‐Estrada, José Francisco Pérez, Sandra van Kuijk, Diego Melo‐Durán, Razzagh Karimirad, David Solà‐Oriol

**Affiliations:** ^1^ Animal Nutrition and Welfare Service Department of Animal and Food Science Universitat Autonòma de Barcelona Bellaterra Spain; ^2^ Trouw Nutrition, Research and Development Department Amersfoort The Netherlands; ^3^ Department of Animal Science Lorestan University Khorramabad Iran

**Keywords:** carcass characteristics, copper and zinc, digestibility, growing pigs, solubility, trace mineral sources

## Abstract

The present study was conducted to evaluate the effect of two Zn supplemented levels and two Zn and Cu sources (sulphate and hydroxychloride) on growing‐finishing pigs. An in vitro study and an in vivo study were conducted. In the in vitro study, Zn solubility from each source at different Zn supplementation levels was evaluated, as well as the phytic phosphorus (PP) solubility derived from the interaction or not with phytic acid at similar conditions to those found in digestive tract. The most critical interaction of Zn with phytic acid was at pH 6.5 and with Zn sulphate, resulting in the reduction in PP solubility. In the in vivo experiment, a total of 444 pigs ([Duroc × Landrace]×Pietrain; initial BW: 18.7 ± 0.20 kg) were allotted to 36 pens in a randomized complete block design (2 × 2) factorial arrangement with two Zn and Cu sources and two Zn supplemental levels (20 and 80 mg/kg). The Cu supplementation was fixed at 15 mg/kg for all diets. There was no effect of the interaction between mineral source × Zn level or Zn level on growth performance or carcass characteristics (*p* > .10). Apparent total digestibility of Zn and Cu along with carcass yield was higher for pigs fed hydroxychloride than pigs fed the sulphate counterparts (*p* < .05). Feeding low levels of Zn decreased Zn (45.5%; *p* < .0001) and Cu(18.5%; *p* = .018) faecal excretion. In conclusion, under commercial conditions, feeding growing‐finishing pigs with Zn levels below those established by the European Union regulation did not affect growth performance and carcass characteristics. Reducing dietary mineral (Zn and Cu) diet content resulted in a lower faecal mineral excretion. Pigs fed sulphate minerals had an improved performance during grower period, while pigs fed hydroxychloride minerals showed an improved performance during finishing period and a greater carcass yield and mineral digestibility than those fed sulphates.

## 1 INTRODUCTION

Trace minerals play an essential physiological role for the normal growth and health of animals. For instance, Zn and Cu are involved in the synthesis of more than 300 enzymes and proteins, immune system activation and bone development, among other functions (Suttle, 2010). The National Research Council (NRC) (2012) established a total requirement of Zn (50–60 mg/kg feed) and Cu (3–4 mg/kg feed) for pigs from 25 to 135 kg body weight (BW). European institutions such as FEDNA (2013) and INRA (1989) have also emitted recommendations for the total feed content of Zn (80 and 100 mg/kg feed) and Cu (8 and 10 mg/kg feed) in growing‐finishing pig diets. However, in non‐European Union (EU) countries (e.g. United States, China, Brazil), the swine industry supplements grower‐finisher diets at higher levels than NRC requirements, such as in United States the supplementation is approximately 1.6 times for Zn and 25.8 times for Cu (Flohr et al., 2016), usually to promote growth (Coble et al., 2017; Liu et al., 2018) and prevent intestinal dysbiosis (Højberg, Canibe, Poulsen, Hedemann, & Jensen, 2005; Liu et al., 2018). At high supplemental, and therefore total dietary levels, trace minerals are barely absorbed, while may interact with other nutrients (Pang & Applegate, 2006; Walk, Wilcock, & Magowan, 2015) or with the activity of other additives, such as phytase (Akter, Graham, & Iji, 2015; Augspurger, Spencer, Webel, & Baker, 2005, 2004). Moreover, due to the efficient homoeostasis systems, when these minerals are supplemented over the animals’ requirements, the mechanisms of absorption are reduced while increasing the endogenous secretions, mainly to intestinal pathways (pancreatic and bile secretions) (Goff, 2018; Spears, 1996; Suttle, 2010). Therefore, large amounts of trace minerals are excreted in manure. Subsequent applications of manure to the soil may increase the ecotoxicity of plants, microorganisms and soil, which is a major environmental concern (EFSA FEEDAP, 2016). Likewise, there is an increasing public health concern due to the association of the great dietary, and therefore environmental, Zn and Cu contents with the increasing microbial resistance rates to antibiotic agents by co‐selection, as well as the possibility of transferring of such resistant bacteria from food‐producing animals to humans such as farmers, veterinarians and consumers (Ciesinski et al., 2018; Yazdankhah, Rudi, & Bernhoft, 2014). To prevent environmental and public health concerns, dietary trace mineral levels can be reduced without affecting the productive performance of pigs. Therefore, EU countries have established maximum total Zn (120 mg/kg) (European Commission, 2016) and Cu (25 mg/kg) (European Commission, 2018) levels for grower‐finisher pigs.

Sulphate mineral sources (35% Zn; 25% Cu) are the most used mineral sources in the animal feed industry due to their high solubility and relatively low cost compared to hydroxychloride and organic bound mineral sources. Sulphates are characterized by having a labile molecular bond that connects the metal ion with the sulphate group, allowing high solubility in water and acid solutions (Guo et al., 2001; Park & Kim, 2016). Therefore, sulphates are frequently used as a reference point for comparing bioavailability of different mineral sources (Guo et al., 2001; Park & Kim, 2016). Alternatively to sulphates, hydroxychloride mineral sources (55% Zn; 54% Cu) have a crystalline structure formed by covalent bonds located between the soluble metal ion, multiple hydroxyl groups and the chloride ions (Zhang & Guo, 2007), with low solubility in water but high solubility in acid solutions (Cao, Henry, Ammerman, Miles, & Littell, 2000). This makes them less reactive with other components in the diet (Lu et al., 2010; Luo et al., 2005). The difference between hydroxychloride mineral sources and chelated or organic counterparts is that the coordinate covalent bond is located between the metal ions and organic molecules (e.g. amino acids, peptides) (AAFCO, 2002).

In the present study, we hypothesized that due to the chemical properties of the trace mineral sources the productive performance of grower‐finisher pigs fed hydroxychloride minerals would be higher than those fed sulphates when Zn and Cu doses are lower than those established by the EU regulations. In order to test the hypothesis, a preliminary in vitro assay and a successive in vivo experiment were conducted. To further understand the Zn solubility from each source and the degree of interaction with phytic acid, as the major binding molecule present in swine diets, the in vitro assay was performed at several Zn concentrations (50, 100, 200 and 300 mg Zn/L) from each source and under similar conditions to those found throughout the gastrointestinal tract (pH 2.5, 4.5 and 6.5). Likewise, the phytic phosphorus (PP) solubility was evaluated. An early similar in vitro assay was performed to assess the Cu solubility characteristics from both mineral sources (Villagómez‐Estrada, Pérez, van Kuijk, et al., 2020). The in vivo experiment was aimed to evaluate the effect of two supplemented Zn levels (20 mg/kg as a low level and 80 mg/kg as a nutritional level) as well as the effect of two Zn and Cu sources (sulphate and hydroxychloride) on growth performance, carcass characteristics, tissue mineral content and mineral apparent total tract digestibility in grower‐finisher pigs reared under commercial conditions. The supplemental level of Cu was fixed at 15 mg/kg for all diets.

## 2 MATERIALS AND METHODS

All animal experimentation procedures were approved by the Ethics Committee of the Universitat Autònoma de Barcelona in compliance with the EU guidelines for the care and use of animals in research (European Parliament, 2010).

### 2.1 In vitro solubility test

The methodology for in vitro test is described in an early in vitro test (Villagómez‐Estrada, Pérez, van Kuijk, et al., 2020) performed to assess the Cu solubility characteristics from both mineral sources. Briefly, the solubility of Zn sulphate, Zn hydroxychloride and the concentration of soluble PP were measured in duplicate. Zn from each source was added at concentrations of 50 mg/L, 100 mg/L, 200 mg/L and 300 mg/L in 200 mM glycine buffer (pH 2.5) (Merck, Germany) and 200 mM sodium acetate buffer (pH 4.5 and 6.5) (Merck, Germany). Zn was mixed with 20 ml of buffer with and without 2.9 mM phytate (Merck, Germany), incubated at 41°C in a shaking water bath for 1 hr and filtered through 42‐μm Whatman filter paper. The solubility of Zn and PP was analysed with inductively coupled, plasma‐optical emission spectroscopy (ICP‐OES, model Optima 4300DV, Perkin‐Elmer Inc.; Waltham; MA, US). The solubility of PP was expressed as mg/L, whereas the solubility of Zn was calculated using the following equation:

$$\text{Mineral Solubility},\%=\left[\left(\text{Soluble mineral}\right)/\left(\text{Total mineral}\right)\right]\times 100$$

### 2.2 In vivo experiment

#### 2.2.1 Animals and Housing

The experiment was performed on a commercial farm in Catalonia, Spain, from February to June with a single batch of pigs. At the end of the nursery phase (63 ± 1.1 d of age), 444 pigs ([Duroc × Landrace] ×Pietrain) purchased from a different commercial farm and with an initial average BW of 18.7 ± 0.20 kg were used in a 105‐d study. Pigs were weaned at 28 ± 1.1 d of age and nursed during 35 d under commercial conditions and fed the same commercial pre‐starter and starter diets. Equal numbers of boars and gilts were ear tag identified and blocked according to sex and initial BW (heavy: 21.7 ± 1.4 kg and light: 15.7 ± 1.9 kg). Pigs were randomly distributed into four experimental diet groups in 36 pens (9 replicate pens/treatment). Pigs were allocated to 12 large pens (10.2 m^2^) of 15 animals and 24 smaller pens (7.5 m^2^) of 11 animals. Each pen had a fully slatted floor and was equipped with a commercial non‐lidded hopper and a nipple drinker to provide ad libitum access to feed and water. The facility was environmentally controlled (temperature and ventilation rate) using thermostatically controlled heaters and exhaust fans.

#### 2.2.2 Experimental design and dietary treatments

Three‐phase diets (Table 1) were formulated to meet or exceed nutrient requirements (NRC, 2012): the pre‐grower phase from d 1 to 21, the grower phase from d 21 to 84 and the finisher phase from d 84 to 105. Four experimental diets were prepared according to a 2 × 2 factorial arrangement, with two Zn and Cu sources (sulphate and hydroxychloride) and two Zn supplementation levels (low: 20 mg/kg, and nutritional: 80 mg/kg). The supplementation level of Cu was fixed at 15 mg/kg for all diets. To achieve these supplemental levels, the total amount of Zn sulphate monohydrate (35%; Pintaluba, Reus, Spain) added was 228.6 g/ton in the nutritional diet, and 57.1 g/ton in the low diet. The supplementary rate of Cu sulphate pentahydrate (25%; Pintaluba, Reus, Spain) was 60.0 g/ton for both sulphate diets. The supplementary amount of hydroxychloride Zn (55%, IntelliBond Z; Trouw Nutrition, the Netherlands) was 145.5 g/ton in nutritional diet and 36.7 g/ton in low diet. The supplemental rate of Cu (54%, IntelliBond C, Trouw Nutrition, the Netherlands) in both hydroxychloride diets was 27.8 g/ton. Phytase was added at 500 FTU per kg of complete feed (Axtra® PHY, Danisco Animal Nutrition, Marlborough, UK). A vitamin‐premix without Zn and Cu was prepared. For each dietary treatment, Zn and Cu products were pre‐mixed with 25 kg of basal diet before being incorporated directly into the mixer during the feed manufacturing process. Finisher phase diets were supplemented with titanium dioxide (0.5%) as an indigestible marker. In order to avoid cross‐contamination with elements from previous productions, feed was manufactured in an appropriate rank order starting with the lower concentrations to be included in the diet. All diets were offered in mash form. Composite samples (1 kg) were collected during the bagging process and were representative of each experimental treatment. Antibiotics or feed additives with antimicrobial properties were not included in the diets.

**Table 1:** Composition of the basal diet for the three phases, as fed‐basis^a^

Ingredients, %	Pre‐growing	Growing	Finishing
Maize	30.00	32.00	32.00
Barley	21.34	13.68	14.65
Wheat	15.01	32.00	32.00
Soya bean meal 47% CP	13.37	12.64	11.99
Extruded soya beans	5.00	‐	‐
Canola meal	‐	5.00	5.00
Carob bean pulp	4.00	‐	‐
Wheat middlings	4.00	‐	‐
Sugar beet pulp	2.50	‐	‐
Lard	1.64	1.77	1.69
DL‐Methionine	0.11	0.07	0.04
L‐Lysine	0.45	0.49	0.46
L‐Threonine	0.16	0.17	0.15
L‐Valine	‐	0.02	‐
L‐Tryptophan	‐	0.02	0.01
Monocalcium phosphate	0.86	0.24	0.21
Calcium carbonate	0.66	0.97	0.91
Sodium bicarbonate	0.15	0.13	‐
Salt	0.35	0.40	0.49
Vitamin‐premix nucleous^b^	0.40	0.40	0.40
Calculated composition			
NE, kcal/kg	2,375	2,400	2,400
CP	16.0	15.8	15.5
Ether extract	4.7	4.0	3.9
Ca	0.60	0.65	0.62
Total P	0.56	0.42	0.42
Dig P	0.35	0.26	0.26
Analysed composition			
CP	15.8	15.5	15.5
Ether extract	4.3	3.4	3.6
NDF	13.0	11.9	12.4
Ash	4.6	5.5	4.1
Ca	0.70	0.78	0.66
P	0.59	0.45	0.40

^a^ Pre‐grower phase diets were fed from d 0 to 21, grower phase diets from d 21 to 84 and the finisher phase diets from d 84 to 105.

^b^ Provided per kg of feed: vitamin A (acetate): 7,150 IU; vitamin D3 (cholecalciferol): 1,320 IU; vitamin E: 25 IU; vitamin K3: 1.10 mg; vitamin B1: 0.88 mg; vitamin B2: 2.75 mg; vitamin B6: 1.10 mg; vitamin B12: 0.02 mg; vitamin B3: 16.5 mg; acid D‐pantothenic acid: 8.80 mg; biotin: 0.01mg; Fe (sulphate): 60 mg; Mn (oxide): 50 mg;; I: 0.55 mg; Se (sodium): 0.27 mg. Butylhydroxytoluene: 0,09 mg; sepiolite: 3.46 mg; phytase: 500 FTU (Axtra® PHY, Danisco Animal Nutrition, Marlborough, UK); glucanase: 1,500 VU and xylanase: 1,100 VU (Rovabio Excel AP, Adisseo, France).

#### 2.2.3 Experimental procedures and sampling

Individual pig weights and feed leftovers were recorded at the end of each feed phase. From these data, average daily gain (ADG), average daily feed intake (ADFI) and gain: feed (G:F) were calculated. In order to reach the market slaughter weight (110 kg), pigs were reared for 25 additional days with the same dietary treatment. During this period, only the individual BW of pigs was recorded one day before slaughter.

At the end of pre‐growing phase, one pig per pen was selected based on the mean BW within the pen (median) to take blood samples by jugular puncture. The same pig was sampled at the end of growing and finisher phases and during slaughter process. Blood samples for determining Zn and Cu were collected into 10‐mL vacutainer tubes (BD Vacutainer®, Z, BD‐Plymouth, UK) that were free of detectable Zn. Serum was obtained after centrifugation (3,000 × *g* for 15 min) and immediately frozen at −20ºC. At d 105, faecal samples were collected from three pigs per pen through digital stimulation, pooled and immediately frozen at −20ºC. At the slaughterhouse, all animals were electrically stunned and killed by exsanguination. Data on the carcass characteristics were collected using an Autofom (SFK, Herlev, Denmark). During the slaughter process, samples of the medial right lobe of the liver (excluding the gall bladder) and right front toe were collected from the selected pig per pen, after weighing the hot carcass and immediately frozen at −20ºC until analysis.

#### 2.2.4 Chemical analysis

Samples of feed were milled at 0.5 mm before chemical and mineral analysis. Analytical determinations of diets were performed according to the AOAC International, (2005, 2004) methods for dry matter (Method 934.01), the Dumas Method for crude protein (Method 968.06), traditional Soxhlet extraction for ether extract (Method 920.39) and ash (Method 942.05). Neutral detergent fibre was analysed using the Ankom nylon bag technique (Ankom 200 fiber Analyzer, Ankom Technology, Macedon, NY).

Liver and faeces were dried in a forced‐air oven at 102ºC for 12 hr, until constant weight, and then milled at 0.5 mm. Bone ash determination protocol was based on the methodology described by Brenes et al., (2003). Briefly, front toe was dissected, and the IV metacarpal was autoclaved for 30 min at 121ºC to remove all the adherent tissues, including cartilage. Subsequently, bone was oven‐dried for 12 hr at 102°C and then soaked in acetone under a chemical hood for 48 hr to extract fat. After this period, the bone was again oven‐dried for 12 hr at 102ºC and weighted before being broken in the middle to be ashed overnight at 550ºC in a muffle furnace. Systematically after the incineration process, bone was again weighed, finely ground with mortar, sieved to a particle size of 0.5 mm and homogeneously mixed before mineral analysis. Bone ash content was calculated based on fat‐free dried bone, and the mineral content is expressed on bone ash basis.

Prior mineral analysis by ICP‐OES samples of feed, faeces, liver and bone ash were digested with concentrated nitric acid (HNO_3_, 65%) in a microwave oven (model Ultrawave, Milestone Srl, Sorisole, Italy). Samples of feed and faeces (0.5 g) were digested with 5 ml of HNO_3_ and 0.2 ml of hydrofluoric acid (HF; 40%) at 240ºC for 30 min. For samples of liver, 0.25 g was digested with 5 ml of HNO_3_ at 220ºC for 15 min; for bone ash samples, 0.20 g was digested with 4 ml of HNO_3_ at 220ºC for 15 min. Serum samples (0.25 ml) were diluted in 1:20 volume ratio with 5 ml of a solution constituted by 0.05% ethylenediaminetetraacetic acid (EDTA) and 0.5% of ammonia (NH_3_). Immediately after digestion or dilution, samples were analysed by ICP‐OES. All samples were analysed for Zn and Cu content.

The coefficient of apparent total tract digestibility (CATTD) of trace minerals was calculated as follow:

$$\text{CATTD}=1‐\left[\left(TiD\times NuF\right)/\left(TiF\times NuD\right)\right]$$

where *TiD* and *TiF* represent the concentrations of the non‐digestible marker in the diet and faeces, respectively; *NuD* and *NuF* represent the nutrient concentrations in the diet and faeces respectively.

### 2.3 Statistical analysis

Data were analysed as a randomized complete block design using the MIXED procedure for mixed linear models of SAS (version 9.4, SAS Institute; Cary, USA). The model included the fixed effects of mineral source, Zn level and their interaction (source × level). Moreover, the block of BW category and sex were considered as random effects. Pen was considered the experimental unit for the performance parameters. The carcass characteristics and mineral tissue content were analysed with individual pig as the experimental unit. The normality and homoscedasticity of data were examined using Shapiro–Wilk test and assessing the normal plot before statistical analysis. When the two‐way interaction between mineral source and Zn level was not significant, the interaction was removed from the model and the data re‐analysed for main factors. Therefore, the main effects are discussed for responses in which the interaction was not significant. Significantly different means were separated using Tukey adjust. Significance was determined at a probability of *p* ≤ .05, and tendencies were considered when the *p*‐value was between > 0.05 and < 0.10.

## 3 RESULTS

### 3.1 In vitro solubility test

**FIGURE 1:**
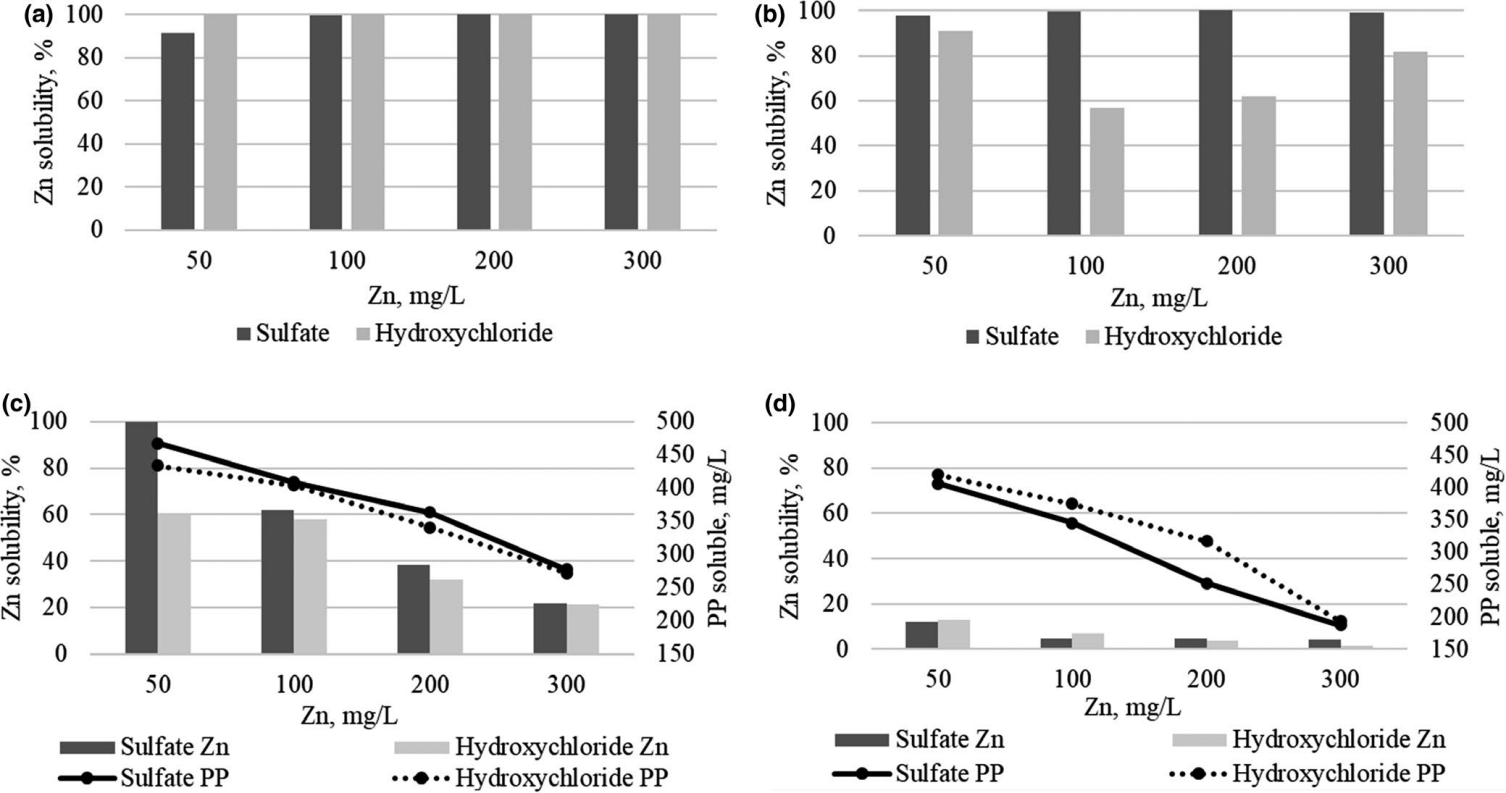
Effects of Zn sourceand level on Zn solubility and soluble phytic phosphorus content at without (a and b) and with (c and d) phytic acid addition at pH 4.5 (a and c) and pH 6.5 (b and d) respectively

The results of Zn solubility sources and their interaction with phytic acid are shown in Figure 1. Overall, the presence of phytic acid decreased the mean Zn solubility as pH increased (from 89% at pH 2.5 to 6% at pH 6.5; Figure 1), regardless of the source. At pH 2.5, the mean solubility of Zn, as an average of the four concentrations, was 100% and 94.7% for Zn hydroxychloride and Zn sulphate respectively (Data not shown). The presence of phytic acid at pH 2.5 reduced the mean solubility of Zn from 97% to 89%, regardless of the source or dose (Data not shown). At pH 4.5, the presence of phytic acid decreased the Zn solubility as the Zn dose increased in both sources (Figure 1c). Phytic acid reduced the Zn hydroxychloride solubility from 100% (without phytic acid) to 43% at pH 4.5 (Figure 1a,c), whereas the mean Zn solubility of sulphate decreased from 98% (without phytic acid) to 55% (Figure 1a,c). At intestinal pH (6.5), the mean solubility of Zn hydroxychloride decreased from 73% to 6% in the presence of phytic acid, while the mean solubility of Zn sulphate decreased from 99% to 6% (Figure 1b,d).

Overall, soluble PP decreased as the pH solution and the Zn dose increased, regardless of the source (pH 2.5 = 424 mg/L; pH 4.5 = 371 mg/L; and pH 6.5 = 312 mg/L; Figure 1c,d). At pH 2.5 and 4.5, the concentration of soluble PP was similar for both sources (Figure 1c). However, at pH 6.5, increasing levels of Zn as sulphate, but not as hydroxychloride, notably reduced the PP soluble content (298 versus 326 mg/L; Figure 1d).

### 3.2 In vivo experiment

#### 3.2.1 Growth performance and carcass characteristics

The analysed mineral content in feed is shown in Table 2. The expected differences between low and nutritional levels of Zn in the diets were achieved with both sulphate and hydroxychloride mineral sources. The Cu content in all diets was within the expected values.

**Table 2:** Analysed trace mineral content in experimental diets

Zn and Cu source	Added Zn, mg/kg	Added Cu, mg/kg	Pre‐grower, mg/kg		Grower, mg/kg		Finisher, mg/kg	
			Zn	Cu	Zn	Cu	Zn	Cu
Sulphate	20	15	54.1	22.0	73.4	25.4	60.7	17.5
	80	15	116.6	30.8	117.6	19.8	115.2	20.5
Hydroxychloride	20	15	50.7	19.6	90.0	22.7	62.0	15.4
	80	15	109.6	20.0	130.0	21.7	97.6	15.7

There was no effect of the interaction between mineral source and Zn supplemented level for any of the growth performance responses (*p* > .10). Therefore, data were re‐analysed and only the main effects of mineral source and Zn supplemented level are presented in Table 3. The Zn supplementation level was not observed to have any effect on the growth performance during the experimental periods (*p* > .10; Table 3). At the end of the grower period, pigs fed Zn and Cu sulphate mineral source had a higher ADG (743 versus 713 g; *p* = .006) and G:*F* (0.524 versus 0.485; *p* = .027) than those fed Zn and Cu hydroxychloride mineral source. However, at the end of the finisher period pigs fed hydroxychloride mineral source diets tended to have a higher ADG than those fed the sulphate counterparts (774 versus 728 g; *p* = .088). No signs of Zn deficiency, like parakeratosis or decreased growth, were observed. No mineral source × Zn supplemented level interaction was observed for any of the carcass characteristics (*p* > .10; data not shown). Likewise, no effect of dietary Zn level was observed (*p* > .10). Pigs fed Zn and Cu hydroxychloride source had a higher carcass yield percentage (*p* < .0001; Table 3) than those fed Zn and Cu sulphate mineral source.

**Table 3:** Growth performance and carcass characteristics of pigs fed diets with two Zn and Cu sources (sulphate and hydroxychloride) at two Zn levels (20 and 80 mg/kg)

Zn and Cu Source	Zn level, mg/kg	Growth performance^a^										Carcass characteristics^b^		
		BW, kg				ADG, g				ADFI, g	G:F	CCW, kg	Lean meat, %	Carcass yield, %
		d 0	d 21	d 84	d 105	d 0–21	d 22–84	d 85–105	d 0–105	d 0–105	d 0–105			
Sulphate		18.87	31.80	78.59	94.06	614.8	743.0	728.4	716.1	1687.1	0.428	74.76	67.13	70.04
Hydroxychloride		18.62	31.44	76.40	92.89	609.9	712.6	773.7	707.2	1734.6	0.409	74.02	67.08	70.57
*SEM*		3.051	3.655	4.584	5.036	29.52	20.61	31.26	21.67	49.40	0.0083	2.022	0.351	0.232
	20	18.66	31.63	77.81	93.91	617.3	730.5	758.2	716.5	1735.2	0.415	73.96	67.26	70.22
	80	18.83	31.61	77.18	93.03	607.4	725.2	743.9	706.8	1686.5	0.421	74.82	66.96	70.40
*SEM*		3.049	3.653	4.579	5.032	29.37	20.54	30.95	21.63	48.72	0.0081	2.039	0.359	0.237
*p*‐*value*														
Source		0.696	0.645	0.086	0.319	0.784	0.006	0.088	0.251	0.325	0.108	0.199	0.816	<0.0001
Level		0.790	0.978	0.608	0.448	0.575	0.611	0.579	0.206	0.312	0.607	0.138	0.165	0.106
Source × Level		0.750	0.841	0.828	0.326	0.363	0.745	0.443	0.281	0.629	0.714	0.479	0.725	0.158

^a^ Data are the average value of *n* = 9 replicate pens for the two‐way interaction, whereas for the main effects of source and level are means of 18 replicate pens.

^b^ Data are the average value of *n* = 444 pigs for the two‐way interaction, whereas for the main effects of source and level are the average value of *n* = 222 pigs. CCW = cold carcass weight.

#### 3.2.2 Apparent total tract digestibility and faecal mineral excretion

**FIGURE 2:**
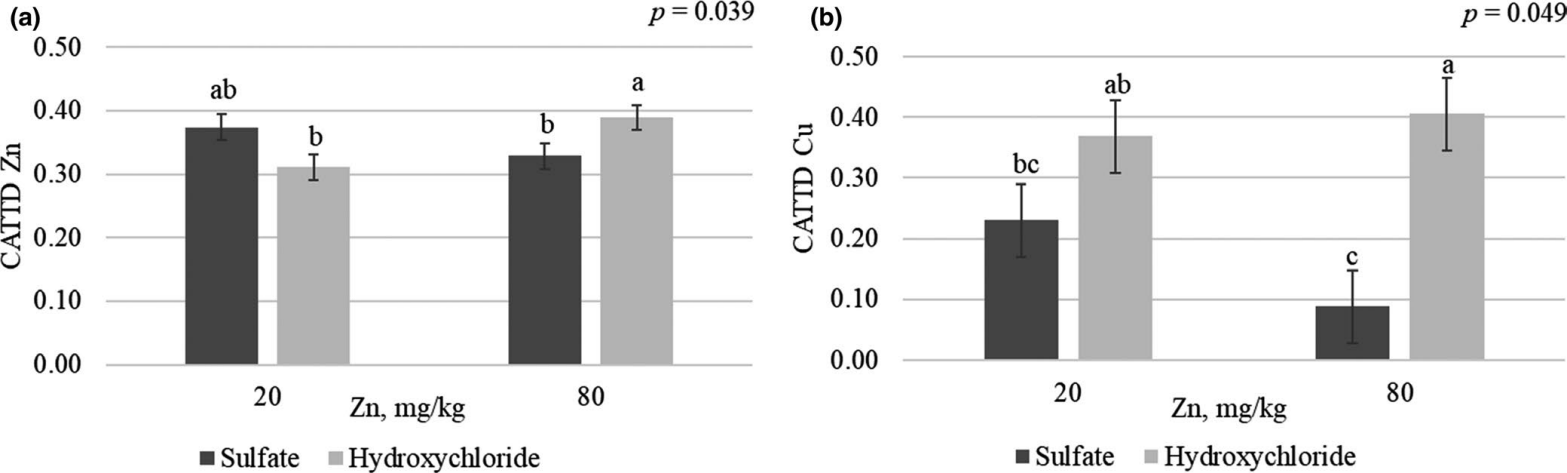
Coefficient of apparent total tract digestibility (CATTD) of Zn (a) and Cu (b) at the end of finisher phase (d 105) of pigs fed diets with two Zn and Cu sources (sulphate and hydroxychloride) at two Zn levels (20 and 80 mg/kg). Data are means of 9 replicate pens for the two‐way interaction (3 pigs per replicate pen were sampled and pooled). a‐c: values with different letters differ significantly for the two‐way source × level (*p* < .05)

A two‐way interaction between mineral source × Zn supplemented level was observed in the CATTD of Zn and Cu (*p* < .05; Figure 2). Pigs fed Zn hydroxychloride at 80 mg/kg had a greater CATTD of Zn (0.39 versus 0.33) and Cu (0.41 versus 0.08) than those fed Zn sulphate at 80 mg/kg, being the nutritional supplemental levels of Zn from both mineral sources intermediate.

**FIGURE 3:**
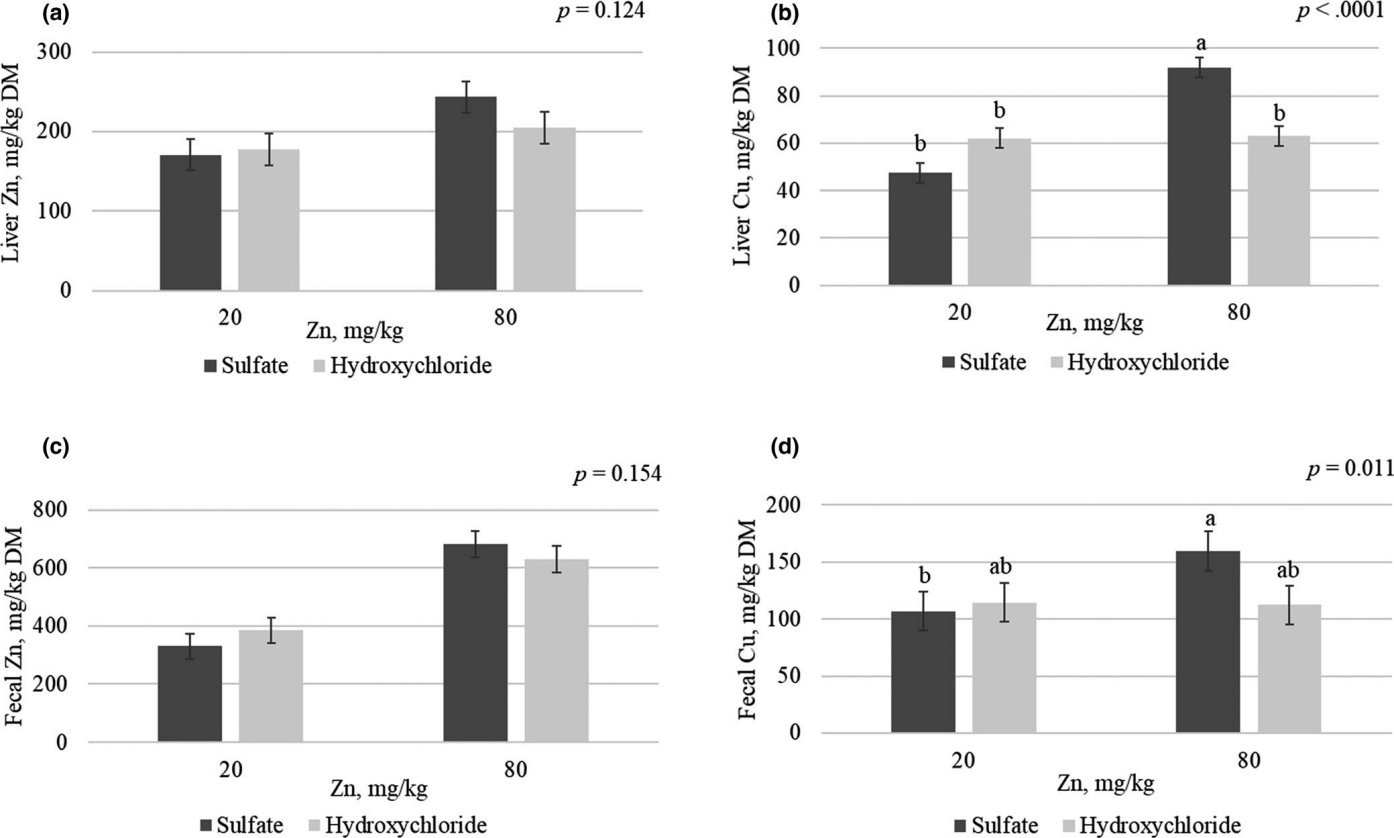
Liver trace mineral accumulation (a‐b) and faecal excretion (c‐d) of pigs fed diets with two Zn and Cu sources (sulphate and hydroxychloride) at two Zn levels (20 and 80 mg/kg). Data are means of 9 replicate pens for the two‐way interaction (1 pig for liver and 3 pooled pig for faeces were sampled per replicate pen). a‐b: values with different letters differ significantly for the two‐way source × level (*p* < .05)

A two‐way interaction mineral source × Zn supplemented level was also observed for Cu faecal excretion (*p* = .011; Figure 3d). Pigs fed Zn sulphate at nutritional supplemental levels excreted higher quantities of Cu than those fed Zn sulphate at low supplemental levels (159.6 versus 107.1 mg/kg), whereas pigs fed Zn hydroxychloride at both nutritional and low supplemental levels were intermediate (112.1 and 114.4 mg/kg respectively). Feeding diets with lower supplemental levels of Zn decreased the Zn (*p* < .0001) and Cu (*p* = .018) excretion by 45.5% and 18.5% respectively (Figure 3c and 3d).

#### 3.2.3 Mineral tissue content

There was no effect of the interaction between mineral source × Zn supplemented level in any of the tissue mineral content except for Cu content in liver (Table 4). Pigs fed Zn sulphate at 80 mg/kg had a higher Cu content (92.0 mg/kg) than pigs with the other experimental diets (*p* < .0001; Figure 3b), while no differences in Zn liver content were observed (*p* > .10; Figure 3a). Feeding diets with Zn at 80 mg/kg increased liver and the metacarpal content of Zn (*p* < .05; Table 4). Likewise, increasing dietary levels of Zn increased the Cu content in the serum and liver (*p* < .01). Pigs fed sulphate trace minerals tended to store higher levels of Cu in the liver than those fed hydroxychloride minerals (*p* = .090). All treatments showed low levels (<20 mg/kg) of Cu storage in the metacarpal bone, below the ICP‐OES detection limit.

**Table 4:** Tissue mineral content of pigs fed diets with two Zn and Cu sources (sulphate and hydroxychloride) at two Zn levels (20 and 80 mg/kg)

Zn and Cu Source	Zn level, mg/kg	Trace mineral content^a^				
		Zn			Cu	
		Serum, mg/L (d 105)	Liver, mg/kg DM	Bone^b^, mg/kg	Serum, mg/L (d 105)	Liver, mg/kg DM
Sulphate		1.07	207.0	219.0	2.02	69.79
Hydroxychloride		1.14	190.7	213.9	1.84	62.50
*SEM*		0.064	16.53	7.83	0.136	2.947
	20	1.10	173.9	204.9	1.84	54.79
	80	1.10	223.8	227.9	2.05	77.50
*SEM*		0.065	16.30	7.62	0.136	2.947
*p‐value*						
Source		0.102	0.226	0.635	0.600	0.090
Level		0.977	0.002	0.038	0.010	<0.0001
Source × Level		0.241	0.124	0.912	0.317	<0.0001

^a^ Data are of the average values of *n* = 9 replicate pens for the two‐way interaction, whereas for the main effects of source and level are the average values of *n* = 18 replicate pens (1 pig per pen was sampled).

^b^ Bone ash basis. Cu‐detected values are lower than 0.02 mg/g by ICP‐OES.

## 4 DISCUSSION

### 4.1 Growing‐finishing pig responses to low dietary Zn levels

The current estimates recommended by European institutions of animal nutrition as well as NRC values for Zn and Cu are considered the minimum requirements for the normal growth of pigs (25–135 kg BW). Contrary to the strategy followed for amino acid, N, Ca and P requirements, which are generated by a modelling approach, the Zn and Cu requirements are derived from a critical evaluation of nutrient requirement studies (NRC, 2012). In accordance with our study, previous studies conducted in growing‐finishing pigs have also shown no differences in growth performance and carcass characteristics between growing‐finishing pigs fed diets with low or non‐supplementary levels of Zn and those fed Zn levels similar or up to NRC requirements (Cemin, Woodworth, et al., 2019; Creech et al., 2004; Gowanlock, Mahan, Jolliff, Moeller, & Hill, 2013; Paboeuf, Nys, & Corlouer, 2000) even under a restricted floor space allowance (Holen, Rambo, Hilbrands, & Johnston, 2018). In general, the requirements for most of the nutrients (as percentage of the diet), including trace minerals, decrease as the BW of the pig increases (NRC, 2012). For instance, higher levels of Zn (100 mg/kg feed) and Cu (6 mg/kg feed) are needed for pigs from 5 to 11 kg BW compared to growing pigs (NRC, 2012). The lack of growth response between these two supplemented levels of Zn in the present study could be attributed to (a) the absence of intestinal and immunological challenges experienced by growing‐finishing pigs. Unlike newly weaned pigs, growing‐finishing pigs have a mature immune system and intestinal microbiome (Wang et al., 2019), which is already adapted to solid feed and other stress factors such as the environmental conditions. (b) The greater intake capacity of growing‐finishing pigs may allow satisfying their Zn requirement as more feed is consumed, although the bioavailability of natural minerals in the cereals and grains of their diet is low or not completely known. (c) The addition of phytase (in the present study at 500 FTU) may have increased Zn bioavailability and minimized other dietary antagonisms of phytic acid with other minerals and digestive enzymes (Revy, Jondreville, Dourmad, & Nys, 2004; Schlegel, Sauvant, & Jondreville, 2013). (d) The supplementation of high‐fibre diets with carbohydrase enzymes (i.e. glucanase and xylanase) may have enhanced the nutrient digestibility, improved intestinal barrier integrity and modulated the intestinal microbiota, consequently, supporting growth performance (Torres‐Pitarch et al., 2020). (e) The complex homoeostasis body system of Zn and Cu may also play an important role in supporting the growth performance of pigs, particularly in those supplemented with a low Zn level, mobilizing minerals from the storage sites in case of being necessary (Suttle, 2010). Nevertheless, it is known that pigs fed with Zn‐deficient diets usually manifest several subclinical and clinical signs such as decreased water and feed intake, growth depression, parakeratosis, hair loss and discoloration. Among these, parakeratosis is the pathognomonic clinical sign and may mark the end point in response to a long‐term insufficient supply of Zn (Brugger & Windisch, 2016). Since in the current study, none of the pigs showed signs of Zn deficiency, and no differences were observed in the long‐term growth performance between Zn dietary levels, it could be suggested that feeding pigs with these low levels did not cause a mineral‐deficit challenge for the animals.

It is not easy to assess the essential trace mineral status of animals, such as Zn and Cu, because the minerals are involved in many physiological functions and their metabolism is complex and not totally understood (Suttle, 2010). Once absorbed Cu from intestine is primarily stored in liver and thereafter distributed, bind to ceruloplasmin, to other tissues such as kidneys, brain, skeleton and skeletal muscle, but in less proportion (Linder et al., 1998; Roberts & Sarkar, 2008). Therefore, liver is considered the main organ responsible for Cu homoeostasis (Goff, 2018). The low Cu values detected in metacarpal bone in the present study may be attributed to the predilection of body for storing greater amounts of Cu in liver than in bone. Generally, the inclusion of pharmacological doses of Zn has been associated with a decrease in Cu bioavailability (Revy et al., 2004). It is likely that, in response to a dietary Zn overload, more metallothionein (MT) proteins are expressed in the enterocytes, which instead bind Cu, reducing its availability to be absorbed (Goff, 2018; Revy et al., 2004). Others have been proposed a competition between Zn and Cu for the use of the divalent metal transporter 1 (DMT1) (Lutsenko, Barnes, Bartee, & Dmitriev, 2007); however, it is also known that both Zn and Cu have their own specific transporters, which are considered the main pathways used to cross the apical membrane (Goff, 2018). Since in the present study, the exposure level of Zn was within the nutritional range, the Cu long‐term storage was not impaired but instead improved as pigs were fed nutritional levels of Zn, suggesting a greater mineral absorption as previously reported (Carlson et al., 2004). Regarding Zn storage in body, as expected, increasing Zn levels in the diets resulted in higher Zn content in target tissues such as liver and bone. The main storages sites of Zn are the liver, muscle and bone (Revy, Jondreville, Dourmad, & Nys, 2003). However, it should be considered that under dietary Zn deficiency the Zn stored in bone cannot be easily mobilized (McDowell, 2003), contrary to others internal pools as liver (Blaabjerg & Poulsen, 2017); therefore, a suitable content of dietary Zn is necessary. In the present study, although pigs fed a low Zn level (20 versus 80 mg/kg) showed lower Zn content in the liver, no differences were observed in growth performance between dietary levels. Nonetheless, complementary studies using additional biomarkers should be considered to confirm that these low dietary supplies match the pigs’ metabolic requirements, especially under stressing or poor sanitary conditions in which the Zn requirements for optimizing the immune function may be higher than those for growth (Klasing, 2001).

Reducing dietary Zn levels resulted in a reduction in Zn and Cu faecal excretion of 45.5% and 18.5% respectively. In agreement with our results, previous studies with pigs have reported that Zn and Cu excretion could be reduced without negative effects on growth performance, for instance, by 50% by reducing micro‐mineral supplementation from commercially utilized levels (Zn 150–100 and Cu 25–15 mg/kg, respectively) to 25 and 5 mg/kg respectively (Creech et al., 2004; Liu et al., 2016; Paboeuf et al., 2000; Van Heugten, O’Quinn, Funderburke, Flowers, & Spears, 2004). The absorption and excretion of Zn and Cu are strictly regulated through homoeostatic control mechanisms, and when these minerals are supplemented over the animals’ requirements, the excess is not absorbed or is endogenously secreted (Goff, 2018; Spears, 1996), ending up in manure. There are increasing environmental and public health concerns about the swine industry due to its contribution to contaminating soils and ground water with high Zn and Cu levels (Wuana & Okieimen, 2011) and possibly spreading antibiotic resistance by co‐selection (Yazdankhah et al., 2014). Therefore, feeding trace minerals to animals as precisely as possible is a potential approach for reducing these environmental and public health risks. The present study found that feeding growing pigs with Zn levels under those established by the EU regulation did not affect growth performance and carcass characteristics, and also resulted in lower faecal mineral (Zn and Cu) excretion.

### 4.2 Growing‐finishing pig responses to different Zn and Cu sources

Interestingly, at the end of the grower feed period, pigs fed diets supplemented with Zn and Cu as sulphate mineral source had higher growth performance than those fed diets with the hydroxychloride counterparts. The opposed was observed in the finisher feed period, where pigs fed hydroxychloride mineral sources diets showed an improved performance than those fed the sulphate sources. Similar effects were described in a meta‐analysis performed by van Kuijk, Jacobs, Smits, and Han, (2019). The authors reported that the supplementation of growing‐finishing pig diets with Zn hydroxychloride (80 mg/kg) resulted in a 3.9% improvement in G:F and ADG, during the last feed phase, compared to Zn sulphate source. It is likely that these improvements, during the last period of pig growth, might be related to improvements in carcass characteristics, as a greater carcass yield in the present study. In agreement, results from previous studies reported that the supplementation of growing‐finishing pig diets with Zn hydroxychloride (50, 100 and 150 mg/Zn kg) promoted greater hot carcass weight (*p* = .041) (Carpenter et al., 2016), higher carcass yield (*p* = .017) and a tendency for greater hot carcass weight (*p* = .058) (Cemin, Carpenter, et al., 2019) compared with the Zn sulphate supplementation. Likewise, the supplementation of Zn (80 mg/kg) as hydroxychloride mineral source resulted in a higher lean meat percentage (*p* = .001) compared with Zn sulphate supplementation (van Kuijk et al., 2019). Similar improvements on carcass composition were reported in a broiler chicken study with the supplementation of Zn and Cu hydroxychloride (Olukosi, van Kuijk, & Han, 2018). One proposed mechanism of action of hydroxychloride minerals is the influence in the ratio of protein to fat deposition (van Kuijk et al., 2019). Nevertheless, Coble et al., (2017) reported no difference in carcass characteristics when pig diets were supplemented with 75 or 150 mg Cu/kg from a sulphate or hydroxychloride mineral source.

Although the metabolic action of Zn and Cu from different sources is not completely understood, results from previous studies reported different beneficial effects of hydroxychloride minerals on animal health, which may influence the beneficial productive results. For instance, the supplementation of weanling pig diets with Cu hydroxychloride decreased the oxidative stress in the duodenum (Fry et al., 2012; Huang et al., 2015) and increased the serum activity of SOD and ceruloplasmin (Zheng et al., 2018) compared to Cu sulphate. Likewise, the supplementation of Cu and Zn hydroxychloride tended to increase serum GPx activity in weaned pigs (Villagómez‐Estrada, Pérez, Darwich, et al., 2020). Although the absorption and retention rates of trace mineral sources were not measured, a greater apparent total tract digestibility was observed for Zn and Cu hydroxychloride, suggesting a better absorption, and therefore less faecal excretion, compared to sulphates. The last could also be related to the solubility characteristics observed throughout the in vitro study and the improvements observed at the end of finisher phase and in carcass characteristics, despite their lower growth performance during grower period.

The results of the present in vitro study of Zn, as well as our previous in vitro study of Cu (Villagómez‐Estrada, Pérez, van Kuijk, et al., 2020), reported that sulphates have a greater range of solubility than hydroxychloride, depending on pH, making them more prone to interact with other components of the diet such as phytic acid. Indeed, the mean Cu solubility for sulphate mineral source reported was of 98% in the whole range of pH levels (from 2.5 to 6.5), whereas the hydroxychloride Cu mean solubility decreased drastically from 100% at pH 2.5 and 4.5 to 9% at pH 6.5. The addition of phytic acid resulted in a decrease in Cu solubility of both sources, being most critical at pH 6.5 and with Cu sulphate than with Cu hydroxychloride, which also resulted in a decreased solubility of PP (Villagómez‐Estrada, Pérez, van Kuijk, et al., 2020), similar to that observed in the present Zn in vitro study. In this sense, different studies of bioavailability in pigs and broiler chickens have reported a slightly higher bioavailability of Zn hydroxychloride (111%) compared with Zn sulphate (Batal et al., 2001) but greater than Zn oxide (159.3%) (Mavromichalis, Webel, Parr, & Baker, 2001; Zhang & Guo, 2007). Similar relative bioavailability of Cu hydroxychloride compared with Cu sulphate has been reported in pigs (112%) and poultry (109%) (Luo et al., 2005; Miles, O’Keefe, Henry, Ammerman, & Luo, 1998). Nevertheless, in the present study no differences between mineral sources in Zn and Cu tissue content were observed, except for the Cu content in liver. Certainly, the mechanisms of the influence on metabolism of Zn and Cu of both trace mineral sources need further research, especially because after a long‐term supplementation of Cu (15 mg/kg), pigs fed Zn sulphate at nutritional levels had a higher Cu content in the liver than the other experimental groups and even higher than the normal range (19 to 56 mg/kg; Puls, 1994). Moreover, besides this greater liver Cu accumulation, a reduced Cu apparent digestibility and a higher Cu faecal excretion were also observed in those pigs. The complex homoeostasis process of Cu is aimed to ensure a constant and sufficient supply of the micronutrient while simultaneously avoiding excess body levels (Scheiber, Dringen, & Mercer, 2013). In this sense, it is possible that to counteract the excessive accumulation of Cu in the liver in pigs fed with a mineral source of Cu sulphate at nutritional levels of Zn, the homoeostasis mechanism reduced the absorption of Cu from the intestine by increasing the sequestration of Cu in MT, while increasing hepatobiliary excretion through the bile into the intestine and, consequently, in the faeces (Bremner, 1998; Gaetke & Chow, 2003). It should be noted that the Cu excreted into bile is poorly available for its reabsorption (Roberts & Sarkar, 2008). Further comprehensive studies involving several complementary analyses need to be performed to elucidate the action mechanisms for hydroxychloride minerals and to understand the trace mineral metabolism better.

In conclusion, feeding growing‐finishing pigs with Zn levels below those established by the European Union regulation did not affect growth performance and carcass characteristics in the present conditions. Moreover, reducing dietary mineral (Zn and Cu) content resulted in a lower faecal mineral excretion, which makes it one of the possible approaches for reducing the associated environmental and public health risks. The supplementation of pig diets with hydroxychloride minerals resulted in higher apparent digestibility of Zn and Cu and carcass yield compared with diets supplemented with the sulphate counterparts. Further studies with complementary physiological biomarkers are necessary to confirm that these low dietary levels match the pigs’ metabolic requirements.

## CONFLICT OF INTEREST

The authors declare no conflict of interest.

## ANIMAL WELFARE STATEMENT

The authors confirm that they have followed European Union standards for the protection of animals used for scientific purposes (European Parliament, 2010).
